# Mechanisms regulating host cell death during *Leishmania* infection

**DOI:** 10.1128/mbio.01980-23

**Published:** 2024-10-11

**Authors:** Juliane C. R. Fernandes, Dario S. Zamboni

**Affiliations:** 1 Department of Cell Biology, School of Medicine of Ribeirão Preto, University of São Paulo, São Paulo, Brazil; Instituto Carlos Chagas, Curitiba, Brazil

**Keywords:** *Leishmania*, macrophages, cell death, apoptosis, pyroptosis

## Abstract

Parasites from the *Leishmania* genus are the causative agents of leishmaniasis and primarily reside within macrophages during mammalian infection. Their ability to establish intracellular infection provides a secure niche for proliferation while evading detection. However, successful multiplication within mammalian cells requires the orchestration of multiple mechanisms that control host cell viability. In contrast, innate immune cells, such as macrophages, can undergo different forms of cell death in response to pathogenic intracellular microbes. Thus, modulation of these different forms of host cell death is crucial for Leishmaniasis development. The regulation of host cell apoptosis, a form of programmed cell death, is crucial for sustaining parasites within viable host cells. Accordingly, several studies have demonstrated evasion of apoptosis induced by dermotropic and viscerotropic *Leishmania* species. Conversely, the prevention of pyroptosis, an inflammatory form of cell death, ensures the establishment of infection by silencing the release of mediators that could trigger massive proinflammatory responses. This manuscript explores how *Leishmania* regulates various host cell death pathways and overviews seminal studies on regulating host cell apoptosis by different *Leishmania* species.

## INTRODUCTION

Leishmaniasis, a complex vector-borne disease, encompasses a spectrum of clinical forms ranging from tegumentary to visceral manifestations. The disease is caused by approximately 20 different *Leishmania* species that infect humans; the global burden of this neglected tropical disease remains significant, with an estimated annual count of 0.7–1 million new cases each year (1). The intricate life cycle of *Leishmania* begins with the transmission of metacyclic promastigotes by infected sandflies, culminating in the differentiation into amastigote form and its establishment within professional phagocytes, especially macrophages. This pivotal interaction between the parasite and host cell sets the stage for a dynamic interaction within the host [reviewed in reference (2)], where the subversion of cell death mechanisms becomes a central theme.

**Fig 1:**
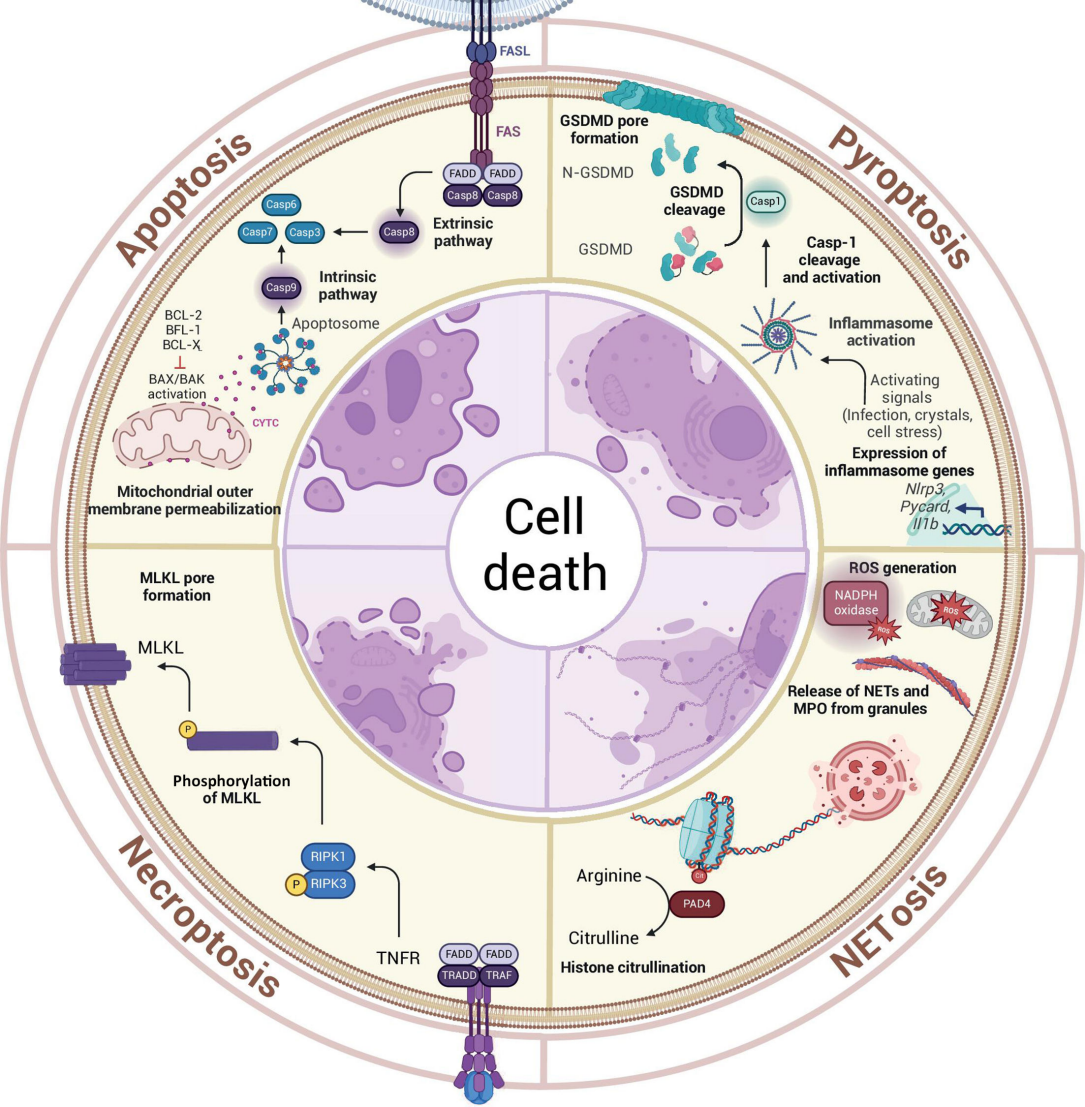
Circular plot illustrating the main signaling pathways and morphological features of cells undergoing apoptosis, pyroptosis, necroptosis, or netosis. Apoptosis is characterized by the activation of Caspase-3, Caspase-6, and Caspase-7, triggered through extrinsic pathways involving Caspase-8 activation by death receptors such as TNFR and FAS or via intrinsic signals leading to Caspase-9 activation via mitochondrial proteins. Pyroptosis involves cleavage of Gasdermin-D (GSDMD) by Caspase-1 or Caspase-11, which are activated by the inflammasome, forming N-terminal GSDMD pores in the membrane, resulting in cell lysis and release of inflammatory contents. Necroptosis is driven by the activation of MLKL, following signaling through RIPK1 and RIPK3, leading to membrane rupture. In NETosis, neutrophils release their DNA and granule proteins, such as myeloperoxidase (MPO), in response to reactive oxygen species (ROS), forming neutrophil extracellular traps (NETs). Each pathway contributes distinct morphological and biochemical hallmarks of cell death, critical for immune responses and tissue homeostasis. Created with BioRender.com

Apoptosis is a type of programmed cell death (PCD) composing a fundamental biological process essential for maintaining the balance and health of multicellular organisms. Apoptosis is a highly regulated mechanism that occurs in response to specific signals or cellular events (Fig. 1). This intricate process involves a series of biochemical events that lead to the controlled dismantling of a cell, preventing the release of harmful substances and ensuring an organized removal without eliciting inflammation. Apoptosis plays a crucial role in various physiological processes, including embryonic development, tissue homeostasis, immune system regulation, and dysregulation of apoptosis, which can contribute to various diseases, including cancer and autoimmune disorders [reviewed in reference (3)]. In the context of host responses against obligate intracellular microbes, apoptosis takes on particular significance. Macrophages, key players in the immune system, can undergo apoptosis as part of the host defense strategy by limiting the replication and spread of intracellular microbes. This controlled death of infected macrophages not only eliminates the host cells harboring the intracellular microbes but also facilitates the efficient clearance of the pathogens by other immune cells. The orchestrated interplay between apoptosis and the immune system thus underscores its crucial role in defending the host against microbial threats and maintaining overall immune homeostasis [reviewed in reference (4)].

**Fig 2:**
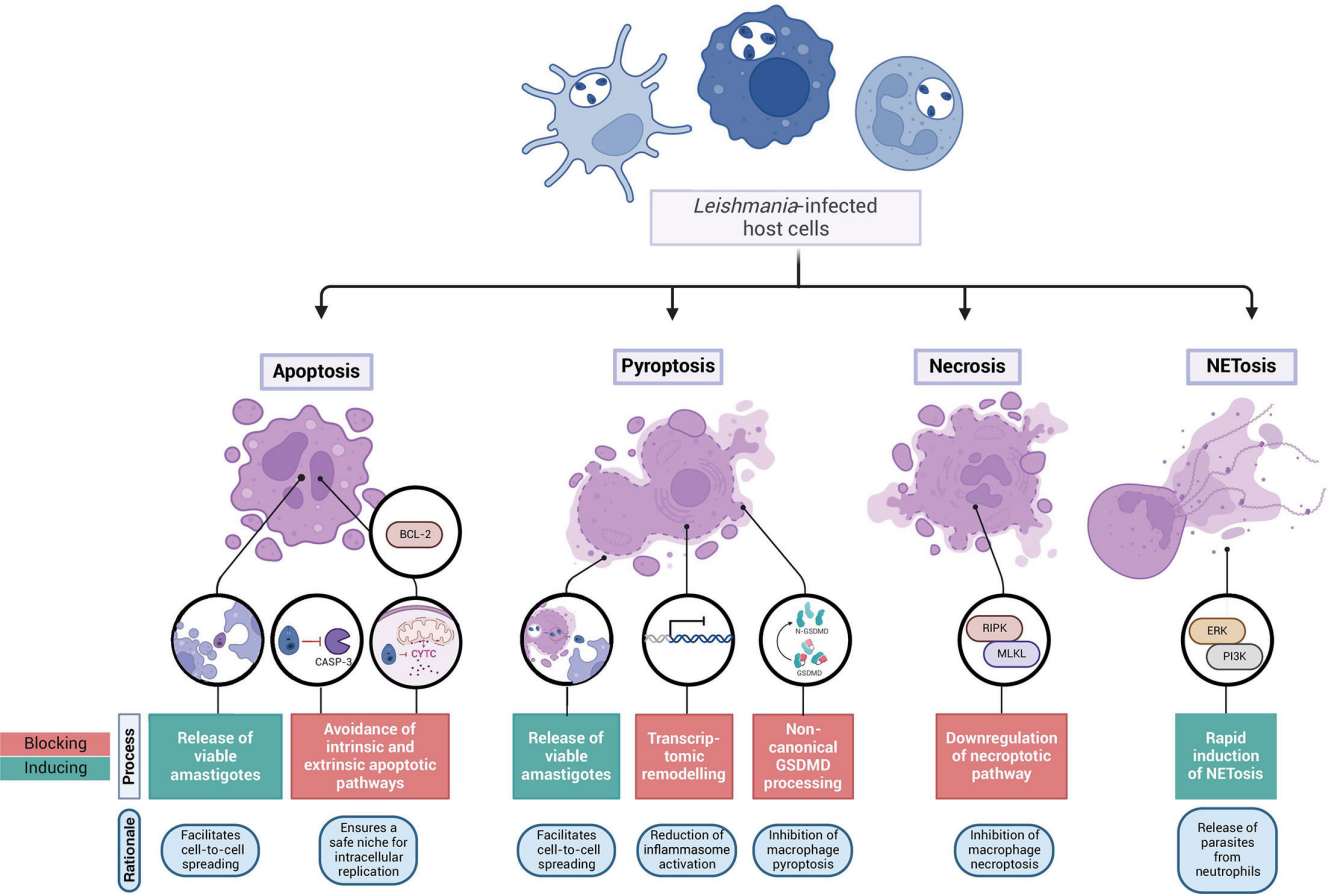
Schematic representation of mechanisms that interfere with host cell death during *Leishmania* infection. *Leishmania* modulates host cell death by either inducing or blocking specific cell death processes. The figure illustrates how *Leishmania* can inhibit apoptosis through the downregulation of pro-apoptotic signals, while in other scenarios, it may induce apoptotic pathways to evade immune detection. In pyroptosis, *Leishmania* can interfere with inflammasome activation, blocking the process to prevent inflammatory cell death, support parasite replication, and inhibit excessive inflammation. The parasite may also inhibit necroptosis, preventing necrotic death to preserve host cells that support parasite replication. *Leishmania* also influences NETosis by regulating the release of neutrophil extracellular traps, putatively promoting this response to favor parasite release from neutrophils. Created with BioRender.com.

In this review article, we explore the intricate and multifaceted aspects of the interplay between *Leishmania* and its host cells, summarizing current knowledge about the complex mechanisms employed by *Leishmania* to manipulate host cell viability (Fig. 2; Table 1), shedding light on the development of Leishmaniasis and potential therapeutic targets for this challenging infectious disease.

**TABLE 1:** Summary of mechanisms used by *Leishmania* to control host cell death

Cell death	Effect	Host cell/species	*Leishmania* species	Reference
Apoptosis	Reduction of M-CSF-induced apoptosis	Macrophage/mice (BMDM)	*L. donovani*	(5)
	Reduction of actinomycin D-induced apoptosis	Monocyte/human (U-937)	*L. infantum*	(6)
	Reduced cytochrome c release	Macrophage/mice (BMDM)	*L. major*	(7)
	Reduction of cycloheximide-induced apoptosis	Macrophage/mice (RAW 264.7)	*L. major* *L. donovani*	(8)
	Decreased neutrophil spontaneous apoptosis decreasing Caspase-3 activation	Neutrophil/mice	*L. major*	(9)
	LPG-dependent delay of apoptosis	Neutrophil	*L. donovani*	(10)
	Delayed apoptosis	Neutrophil/human (peripheral blood)	*L. major*	(11)
	Reduction of camptothecin-induced apoptosis	Dendritic cell (moDCs)	*L. mexicana*	(12–15)
	Reduction of apoptosis by inhibition of cytochrome c release	Neutrophil/human (peripheral blood)	*L. major*	(16)
	Reduction of apoptosis by inhibition of cytochrome c release	Macrophage	*L. donovani*	(17)
	Reduction of actinomycin D-induced apoptosis	Monocyte/human (U-937)	*L. infantum*	(18)
	Reduction of camptothecin- and actinomycin D-induced apoptosis	Macrophage/mice (BMDM)	*L. major*, *L. pifanoi,* and *L. amazonensis*	(19)
	Reduction of apoptosis	Macrophage/mice (BMDM and RAW 264.7)	*L. donovani*	(20–23)
	Reduction of CSF-1 depletion- and actinomycin D-induced apoptosis	Macrophage/mice (BMDM)	*L. amazonensis*	(24)
	Induction of apoptosis	Macrophage/mice (peritoneal)	*L. major*	(25)
	Induction of apoptosis	Macrophage/mice (BMDM and RAW 264.7)	*L. amazonensis*	(26)
	Induction of apoptosis	Macrophage/mice (peritoneal)	*L. amazonensis*	(27)
	Induction of apoptosis	Macrophage/mice (BMDM) and human (hMDM) Skin lesions	*L. major*	(28)
Pyroptosis	Suppression of pyroptosis	Macrophage/human (U-937 and THP-1)	*L. donovani*	(29)
	Pyroptosis-dependent spread of infection	Macrophage/mice (BMDM)	*L. amazonensis*	(30)
	Suppression of pyroptosis	Macrophage/mice (BMDM) and *ex vivo Leishmania-*infected macrophages (*ev*LIMs)	*L. amazonensis*	(24)
	GSDMD-dependent infection	Monocyte/human (BLaER1-derived monocytes)	*L. major*	(31)
	Suppression of pyroptosis	Macrophage/mice (BMDM)	*L. amazonensis*	(32)
Necroptosis	Heme-dependent induction of necroptosis	Macrophage/mice (BMDM)/human (THP-1)	*L. infantum*	(33)
	Neutrophil necrosis-dependent parasite clearance	Neutrophil/mice (peritoneal) and human (primary)	*L. infantum*	(34)
NETosis	NET release	Neutrophil	*L. amazonensis, L. infantum,* and *L. donovani*	(35–39)

## *LEISHMANIA* SUBVERSION OF HOST CELL APOPTOSIS AS AN EVASION STRATEGY

Apoptosis is characterized by cell shrinkage, membrane blebbing, disturbance of the cytoskeleton, and nuclear condensation without plasma membrane disruption. In addition to serving as a vital mechanism for maintaining cell homeostasis, apoptosis is a defense mechanism against intracellular pathogens [reviewed in reference (4)]. Regarding this, pathogen-host cell coevolution has given rise to multiple regulatory roles controlling apoptosis upon infection.

As an intracellular microbe that infects and replicates in macrophages, *Leishmania* was shown to subvert macrophage apoptosis to maintain its replicative niche. The first demonstration that *Leishmania* infection can modulate host cell apoptosis was published in 1994, showing that *L. donovani* infection or lipophosphoglycan (LPG) treatment could inhibit apoptosis induced by M-CSF depletion in bone marrow-derived macrophages (BMDMs) (5). Likewise, *L. infantum* infection, LPG treatment, or *Leishmania* supernatant inhibited actinomycin D-induced apoptosis of the U-937 human monocytic cell line (6). Subsequent investigations revealed that *L. major* could similarly prevent apoptosis in BMDM from both susceptible BALB/c and resistant C57BL/6 mouse models, indicating that prevention of apoptosis is an intrinsic parasite-induced mechanism reducing cytochrome c (CYTC) release independent of host genetic background (7). Collectively, these studies proposed the induction of anti-apoptotic mechanisms by both dermotropic and viscerotropic *Leishmania* species. Further investigation has revealed that *Leishmania*’s ability to prevent cycloheximide-induced apoptosis depends on parasite strain. It was demonstrated that *L. major* V1, Spock, and IR173 prevent apoptosis to a much greater degree than LV39 and NIH S strains, while the *L. donovani* 1S strain presents a higher capability to prevent apoptosis than 9515 and Mongi strains (8). Whether these differences account for disease severity by these strains remains to be investigated.

Neutrophils are usually short-lived cells that are the initial host cells encountered by *Leishmania* upon mammalian host infection (40). Phagocytosis of viable *L. major* delays spontaneous apoptosis of neutrophils both *in vitro* and *in vivo* by decreasing Caspase-3 activation (9). This mechanism was also observed in *L. donovani*-infected neutrophils, while phagocytosis of parasites lacking LPG (*lpg2^−/^*^−^) could not increase neutrophil longevity (10), again suggesting that LPG is involved in the modulation of host cell death. Apoptotic cells emit signals that prompt rapid phagocytosis by neighboring phagocytic cells without eliciting a pro-inflammatory response, serving as a mechanism facilitating parasite establishment [reviewed in reference (4)]. Neutrophils harboring *Leishmania* attract macrophages to the infection site by secreting the chemokine Macrophage Inflammatory Protein (MIP)-1β (11). Also, infection of monocyte-derived DCs (moDCs) with both *L. mexicana* promastigotes and amastigotes resulted in a decrease in camptothecin-induced apoptosis. The increase in cell viability was accomplished by the reduction of Caspase-3 activation (12–14).

## MOLECULAR MECHANISMS INVOLVED IN *LEISHMANIA* INTERFERENCE ON APOPTOTIC SIGNALING PATHWAYS

Different apoptosis-activating pathways result in controlled activation of cysteine proteases, the caspases, which are synthesized as inert pro-forms. Autocatalytic activation of Caspase-9 occurs during intrinsic apoptosis, while extrinsic apoptosis initiates with Caspase-8 activation (Fig. 1). Both caspases mediate the activation of the executioner Caspase-3, Caspase-6, and Caspase-7, which are the effectors promoting the apoptotic phenotype, such as DNA fragmentation and phosphatidylserine (PS) exposure in the outer plasma membrane [reviewed in reference (41)]. The most prevalent triggers of caspase-activating cascades are natural killer- and T-cell-secreted granzyme and perforin, extrinsic pathway depending on ligands of death receptors of the TNF family [such as Fas cell surface death ligand (FasL) and the TNF superfamily member 10 (TNFSF10)], and the intrinsic pathway, depending on the release of mitochondrial CYTC triggered by deprivation of growth factors, DNA damage, reactive oxygen species (ROS) production, endoplasmic reticulum (ER) stress, and others [Fig. 1 and reviewed in reference (41)]. CYTC release depends on mitochondrial outer membrane permeabilization mediated by pro-apoptotic proteins from the B-cell lymphoma 2 (BCL2) family, such as BCL2 associated X (BAX), BCL2 antagonist/killer 1 (BAK) and BCL2 family apoptosis regulator (BOK), while BCL-2, BCL2-related protein A1 (BCL2A1 or BFL-1), BCL2 like 1 (BCL2L1 or BCL-X_L_), and BCL-2 interacting mediator of death (BIM) antagonize this process [Fig. 1 and reviewed in reference (41)].

Neutrophil infection with *L. major* induces upregulation of antiapoptotic Bcl-2 and Bfl-1, activating p38 Mitogen-activated Protein Kinase (MAPK) and extracellular signal-regulated kinase 1/2 (ERK1/2) pathways, but not AKT, resulting in inhibition CYTC release (16). *L. donovani*-infected macrophages present an upregulation of myeloid cell leukemia 1 (MCL-1) via the induction of the transcription factor CREB. This, in turn, impedes BAK-mediated CYTC release, thereby inhibiting apoptosis (17). Further corroborating these findings, *L. infantum* infection protected human U-937 cells from actinomycin D-induced apoptosis in a Bcl-2- and inhibitor of apoptosis IAP1/2-dependent manner (6, 18). It was also demonstrated that Bcl-2 is increased in peripheral blood monocytes from visceral leishmaniasis (VL) patients compared to healthy controls (HCs). The increase in Bcl-2 was also implicated in preventing nitric oxide (NO) production in mouse peritoneal macrophages upon *L. donovani* infection, while inhibiting this protein reduced parasite load both *in vitro* and *in vivo*. This finding presented novel functions for a known major regulator of apoptosis (42).

Several studies demonstrated the role of PI-3 kinase (PI-3K)/Akt pathways in regulating cell survival (43). Indeed, *L. major*, *L. pifanoi*, or *L. amazonensis* infection activated these pathways, reducing apoptosis by apoptosis-inducing stimuli (19). Activation of PI-3K/Akt also occurs in DCs infected with *L. mexicana* amastigotes (15). This finding was corroborated in *L. donovani* infection, demonstrating decreased parasite survival and increased macrophage apoptosis *in vitro* by treatment with AKT inhibitors and liver and spleen parasite burden *in vivo* (21). Mechanistically, further studies demonstrated that AKT activation and inhibition of apoptosis depends on programmed death-1 receptor (PD-1) downregulation by *L. donovani* (22). It was shown that AKT regulation was also dependent on the cellular response to stress upon *L. donovani* infection. The parasite induces ER stress, resulting in the activation of the unfolded protein response (UPR) and the PKR-like ER kinase (PERK) results in phosphorylation and activation of AKT in murine RAW macrophages, which in turn mediates delayed apoptosis (23). *Leishmania*-induced activation of AKT mediates inhibition of apoptosis by phosphorylation of glycogen synthase kinase 3β (GSK-3β) and transcriptional modulation mediated by β-catenin (21). Also, *L. donovani* infection induces cytokine signaling suppressors (SOCS), important regulators of ROS-elicited apoptosis through protein-tyrosine phosphatases (PTPs) activity, leading to decreased apoptosis of infected macrophages despite the oxidative burst during infection (20).

A preprint article demonstrated that *L. amazonensis* amastigotes-infected macrophages resist apoptotic signals of both intrinsic and extrinsic pathways by both CSF-1 deprivation and actinomycin D treatment, respectively (24). Likewise, infection prevented LPS/ATP-induced pyroptosis (to be further discussed in the next topic). This led to an increased *in vitro* macrophage lifespan of over 50 days with over 70 amastigotes per macrophage in large vacuoles that resembled those obtained from infected tissue of mice (24).

## BENEFICIAL EFFECTS OF HOST CELL APOPTOSIS FOR *LEISHMANIA* INFECTION

Interestingly, multiple studies have also shown that the promotion of apoptosis may be beneficial for *Leishmania* infection. It was demonstrated that Fas-L-mediated apoptosis of neutrophils is important for *L. major* maintenance in susceptible Balb/c mice. This process exacerbates parasite replication in neutrophils that are attracted to the site of infection by apoptotic resident macrophages (25). The Fas-L-dependent increased apoptosis of neutrophils upon *L. infantum* infection was further enhanced by its vector, *Lutzomyia longipalpis*, salivary components. Apoptosis induction correlated with increased parasite burden in neutrophils, while inhibiting caspases *in vitro* abolished *L. infantum* replication. However, apoptosis is not the only immunomodulatory mechanism present in the saliva, and the phenotype may be a consequence of the indirect effects of the induction of the COX-2-derived PGE2 and decreased ROS production (44).

*L. amazonensis* cell-to-cell spreading is also mediated by apoptotic cells (26). In addition, it was suggested that increased virulence of *L. amazonensis* over *L. guyanensis* in both C57BL/6 and BALB/c mice depended on the ability of amastigotes to infect new cells by apoptosis induction (27). According to these reports, it was recently shown by intravital microscopy of skin lesions that *L. major* induces apoptotic cell death as a transferring mechanism between host cells without exposure in the extracellular milieu (28). This finding shows that the induction of apoptosis may be an important mechanism for infecting new cells. Also, this study demonstrates that apoptosis is induced by rapidly proliferating parasites, while parasites can maintain for more extended periods without inducing host cell apoptosis by reducing proliferation rates (28). Despite these reported beneficial effects of host cell apoptosis for *Leishmania* infection, the specific contexts in which host cell apoptosis is beneficial or detrimental to parasite establishment and survival are still to be clarified.

## *LEISHMANIA* SUBVERSION OF MACROPHAGE PYROPTOSIS

Pyroptosis is a form of programmed cell death characterized by rapid cell lysis and the release of pro-inflammatory contents, typically in response to infection or cellular damage. The process usually occurs in response to inflammasome activation, which leads to activation of inflammatory caspases that in turn promotes the cleavage of a pore-forming protein called gasdermin-D (GSDMD). Among the inflammatory caspases involved in GSDMD cleavage are Caspase-1, Caspases 4 and 5 in humans, and Caspase-11 in mice. They promote a GSDMD cleavage generating a 30 kDa N-terminal domain, which promotes pore formation in the plasma membrane mediating IL-1β release, ion exchange, cell swelling, and cell death (Fig. 1) [reviewed in reference (45)]. However, while it has been shown by multiple groups that *Leishmania* infection induces NLRP3 inflammasome activation [reviewed in reference (46)], the repertoire of studies exploring modulation of pyroptosis by *Leishmania* remains relatively limited when compared to apoptosis.

Dampening of pyroptosis via reduction of macrophage transcriptional activities was demonstrated upon *L. donovani* infection; transcriptional reduction of caspase-1, NLRP3, and other inflammasome-related genes was observed in PBMCs from VL patients and in macrophage-infection *in vitro* (29). Mechanistically, *L. donovani* positively regulates the transcription factor B lymphocyte-induced maturation protein 1 (BLIMP-1), which suppresses macrophage pyroptosis by impairing the NF-κB-NLRP3 activation pathway (29).

Another study reported that *L. amazonensis-*infected macrophages reduced pyroptosis after NLRP3 activation with LPS/ATP both *in vitro* and *ex vivo*. This effect was demonstrated at the level of transcriptomic changes upon infection, with a reduction of the *Nlrp3*, *Casp1*, *Pycard*, *Il1b*, and *Il18* transcripts of inflammasome activation pathway in infected macrophages (24). Interestingly, a previous study demonstrated that BMDMs do not require priming and transcriptional regulation of inflammasome components NLRP3, ASC, and CASP1 for the inflammasome assembly upon *L. amazonensis* infection (47). These data may explain the reported inflammasome activation in response to *Leishmania* infection despite the inhibitory activity of *Leishmania* on macrophage proinflammatory gene expression (24, 48–50).

Regardless of the magnitude of inflammasome activation in macrophages infected with *Leishmania*, it is clear that inflammasomes are active in response to infection [reviewed in reference (46)]. Interestingly, although *Leishmania* infection in macrophages promotes inflammasome activation, *Leishmania*-infected macrophages remain viable, and pyroptosis does not occur. This process was recently investigated, and the mechanisms involve an alternative cleavage of GSDMD. It was demonstrated that the multiple species of the parasites induce non-canonical processing of GSDMD into a 25 kDa fragment (opposed to the canonical 30 kDa fragment). This alternative cleavage prevents the canonical pore formation that mediates pyroptosis (32). The specific proteases involved in the non-canonical cleavage have yet to be determined, but the fact that host proteases, including Caspase-1, -7, -8, and -11, are dispensable for the non-canonical GSDMD cleavage, suggesting that a parasite protease may be involved in this process. Alternatively, a recent report indicated that Caspase-3 was involved in generating an inactive 23 kDa fragment of GSDMD (51). Whether Caspase-3 is involved in the non-canonical GSDMD fragment observed in response to *Leishmania* is a matter for future investigation. Despite the alternative cleavage of GSDMD by *Leishmania*, this molecule is still important for protective immune responses against the parasites. Mice and macrophages deficient in *Gsdmd* are highly susceptive to infection with *L. major*, *L. amazonensis*, *L. mexicana*, *and L. braziliensis*, indicating that GSDMD neutralization by *Leishmania* is not complete and the molecule still contributes to host resistance (32). This work also reports active GSDMD in skin biopsy of patients with cutaneous leishmaniasis, supporting a key role of GSDMD in human Leishmaniasis (32).

Regardless of the parasite’s inhibitory activity in GSDMD, a study used real-time imaging to evaluate pyroptosis using real-time imaging on *L. amazonensis*-infected BMDMs treated with LPS priming and ATP. It was shown that upon cell death induced by LPS + ATP, viable amastigotes remain tethered to the parasitophorous vacuole membrane, retaining the capability to infect other macrophages (30). Furthermore, using an *in vitro* model employing BLaER1-derived monocytes, the depletion of GSDMD was demonstrated to prevent the release of *L. major*. The study demonstrated that pyroptosis induction of infected macrophages with LPS and nigericin released viable parasites that infected non-stimulated macrophage posteriorly added to the culture (31), Collectively, these data suggest that even though *Leishmania* inhibits pyroptosis, in case it happens either via GSDMD-mediated pyroptosis or by a CD8-mediated induction of macrophage cell death via perforin and granzyme/granulysin (52), *Leishmania* may bypass this natural host defense and remain able to infect additional macrophages. The interference of *Leishmania* in the GSDMD pathway and pyroptosis has only recently been investigated, and further investigation of this pathway may reveal important aspects for our understanding of complex mechanisms involved in *Leishmania*-macrophage interaction and its importance for the outcome of Leishmaniasis.

## *LEISHMANIA* MANIPULATION OF ADDITIONAL FORMS OF HOST CELL DEATH

Besides the abovementioned mechanisms of host cell death, some studies also demonstrated that *Leishmania* modulates necroptosis, which is also an inflammatory form of cell death characterized by rapid cell swelling and lysis. Necroptosis is regulated by key serine/threonine kinases such as receptor-interacting protein kinases 1 and 3 (RIPK1 and RIPK3) and the mixed lineage kinase domain-like (MLKL), which form pores in the plasma membrane (Fig. 1) [reviewed in reference (53)]. Initial speculations about necroptosis during Leishmaniasis derived from observations that VL patients present higher plasma levels of LDH compared to HC accompanied by classic neutropenia, leading to the interrogation of the mechanisms involved in leukocyte death. *In vitro* experiments using primary human and mouse neutrophils detected RIPK1-RIPK3- and MLKL-dependent necroptosis of neutrophils upon *L. infantum* infection when Caspase-8 is inhibited, along with this pathway requirement to limit *Leishmania* infection in neutrophils (34). Also, high levels of heme in the plasma of VL patients led to the interrogation and identification of a mechanism involving heme-mediated stimulation of necroptosis and *Leishmania* control *in vitro*, while demonstrating the key role of RIPK1 in controlling infection *in vivo* (33)

More evidence at the transcription level points to necroptosis inhibition by dermotropic *Leishmania* species, but whether this type of cell death presents clinical relevance in cutaneous Leishmaniasis remains to be investigated. It was demonstrated that RIPK3 expression is reduced in skin biopsies of patients infected with *L. braziliensis* (54). Several transcripts were also deregulated upon *L. amazonensis* infection of murine macrophages, consistent with an anti-necroptotic signature, including downregulation of *Ripk1*, *Ripk3*, *and Mlkl* and upregulation of *Tnfaip3* (24). Whether this results from a global transcriptional inhibition induced by *L. amazonensis* or a response specific to target host cell necroptosis is a matter for future investigation.

NETosis is a type of neutrophil cell death resulting in the extracellular release of DNA traps called NETs. The induction of ROS, produced either by mitochondria or NADPH, inhibits actin polymerization. This destabilization leads to the release of neutrophil elastase (NE) and myeloperoxidase (MPO), which culminates in histone cleavage. Additionally, PAD4-mediated histone citrullination promotes chromatin decondensation. Both processes induce the extracellular release of DNA (Fig. 1). It was demonstrated that *L. amazonensis*, *L. infantum*, and *L. donovani* infection rapidly induce NETosis (35–38). The induction is mediated by ERK/PI3K pathway and presented early redox-independent mechanisms, while it was sustained at later stages by induction of ROS (38, 39). Although LPG is not responsible for signaling for induction of NET formation, it confers resistance to leishmanicidal activity of NETs (35). Also, *L. infantum* avoids killing by NETs by its 3′ nucleotidase/nuclease (37). Formation of NET structures was confirmed in biopsies of cutaneous Leishmaniasis lesions (36) and NET-related proteins were found in sera of VL patients (55), confirming the induction of this type of host cell death during Leishmaniasis. More investigation will be required to investigate if *Leishmania* evolved specific strategies to manipulate NET formation during infection.

## CONCLUSIONS AND PERSPECTIVES

The numerous reports of manipulation of host cell death by different *Leishmania* species highlight these pathways as promising targets for developing host-directed therapy for Leishmaniasis treatment. However, which parasite factors drive changes in host cell signaling remains largely unknown. Identification of such factors may also reveal suitable targets for anti-*Leishmania* therapy. The demonstration of an induction of an anti-cell death transcriptional landscape, mitigating apoptosis, pyroptosis, and necroptosis, underscores the significance of transcription factors as promising targets for interfering with *Leishmania*’s rewiring of host cells.

Another point that requires additional investigation is related to *in vivo* studies. While most compiled studies primarily focus on mechanisms *in vitro*, the complexity of the role of cell death during Leishmaniasis *in vivo* requires a more comprehensive study using animal models of infection and clinical samples. Despite a recent study proposing let-7a microRNA inhibitors as an effective tool to enhance apoptosis and necroptosis in *Leishmania*-infected macrophages (56), we still lack evidence regarding the role of cell death at each stage of the disease and the contribution of each form of cell death in different cell types participating in *Leishmaniasis* immunopathology. Moreover, while inhibiting host cell death is important for maintaining intracellular replication niches, the induction of host cell death and membrane rupture at some point of infection may be necessary for parasite spreading. In this context, the recently identified mechanisms of *Leishmania* spread upon pyroptotic host cell death (31) add to the complexity of understanding the interplay between the parasite and host cells. Further investigation in this area may reveal important aspects for understanding the interaction of *Leishmania* with its host cells and its role in the development of Leishmaniasis.
